# Elevated plasma myoglobin level is closely associated with type 2 diabetic kidney disease

**DOI:** 10.1111/1753-0407.13508

**Published:** 2023-11-30

**Authors:** Lin Yang, Yan Shen, Wenxiao Li, Bingbing Zha, Wenjun Xu, Heyuan Ding

**Affiliations:** ^1^ Department of Nephrology, Shanghai Fifth People's Hospital Fudan University Shanghai China; ^2^ Department of Endocrinology, Shanghai Fifth People's Hospital Fudan University Shanghai China; ^3^ Center of Community‐Based Health Research Fudan University Shanghai China; ^4^ Jiangchuan Community Health Service Center Shanghai China; ^5^ Department of Nephrology Zhejiang Kaihua County Hospital of Chinese Medicine Zhejiang China

**Keywords:** glomerular filtration rate, inflammation, nephropathy, oxidative damage, type 2 diabetes

## Abstract

**Background:**

Diabetic kidney disease (DKD) is the most frequent complication in patients with type 2 diabetes mellitus (T2DM). It causes a chronic and progressive decline in kidney function, and ultimately patients require renal replacement therapy. To date, an increasing number of clinical studies have been conducted to explore the potential and novel biomarkers, which can advance the diagnosis, estimate the prognosis, and optimize the therapeutic strategies at the early stage of DKD. In the current study, we sought to investigate the association of plasma myoglobin with DKD.

**Methods:**

A total of 355 T2DM patients with DKD and 710 T2DM patients without DKD were enrolled in this study. Laboratory parameters including blood cell count, hemoglobin A1c, biochemical parameters, and plasma myoglobin were recorded. Patients were classified on admission according to the tertile of myoglobin and clinical parameters were compared between the groups. Pearson correlation analysis, linear regression, logistic regression, receiver operating characteristics (ROC) analysis, and spline regression were performed.

**Results:**

Plasma myoglobin significantly increased in patients with DKD and was associated with renal function and inflammatory parameters. Plasma myoglobin was an independent risk factor for the development of DKD. The area under ROC curve of myoglobin was 0.831. Spline regression showed that there was a significant linear association between DKD incidence and a high level of plasma myoglobin when it exceeded 36.4 mg/mL.

**Conclusions:**

This study shows that elevated plasma myoglobin level is closely associated with the development of kidney injury in patients with T2DM.

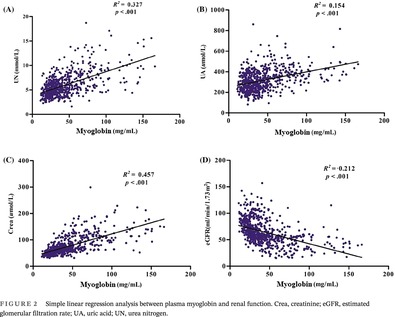

## Graphical abstract

**Highlights**

- Plasma myoglobin significantly increased in patients with diabetic kidney disease (DKD).
- Plasma myoglobin was an independent risk factor for DKD.
- Plasma myoglobin serve as a marker to predict DKD.

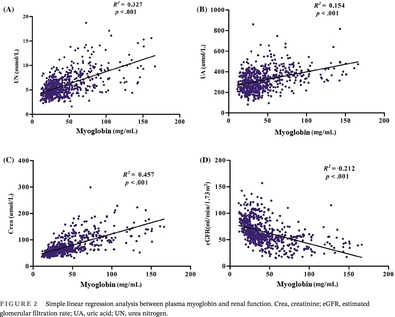

## 1 INTRODUCTION

Diabetic kidney disease (DKD), known as diabetic nephropathy, is the most frequent complication of type 2 diabetes mellitus (T2DM).^1^ In the United States, over 40% of individuals with T2DM develop to DKD.^2^ To date, DKD is considered as a chronic progressive disease involving the whole kidney and becomes one of the predominant causes of chronic kidney disease and end‐stage renal disease worldwide.^3^ DKD causes multiple symptoms and a reduced quality of life. Due to progressive decline in kidney function, the patients finally have to receive renal replacement therapy. Meanwhile, the high prevalence, disability, mortality, and long‐term treatment of DKD have brought huge financial and medical burdens to the whole society and individuals.^4^, ^5^ Thus, the diagnosis and treatment of DKD are standardized, but more potential and novel biomarkers need to be explored.

Myoglobin is an iron‐containing protein. It is synthesized in cardiomyocytes and skeletal muscle cells and plays an important role in the storage and transport of molecular oxygen for cellular respiration.^6^, ^7^, ^8^, ^9^, ^10^, ^11^ In a cross‐sectional study, a chronic subclinical increase in myoglobin was observed in patients with diabetes,^12^ which may be related to increased muscle oxygen consumption and muscle damage.^13^, ^14^

Myoglobin is also an antioxidant with peroxidase activity, which protects cells by eliminating reactive oxygen species (ROS) during cellular hypoxic.^15^, ^16^, ^17^ The prolonged incubation of myoglobin with glucose produces fructosamine, followed by the formation of advanced glycation end‐products (AGEs).^18^, ^19^, ^20^ AGEs were closely associated with renal failure in T2DM and high‐risk kidney disease. Compared with low AGE patients, high AGE patients had a sustained 30% decline in renal function and a significantly increased risk of high‐risk kidney disease.^21^ The increased production of AGEs associated with diabetes is commonly reported as a central cause of diabetic microvascular and macrovascular complications.^22^, ^23^ A recent machine learning‐based study suggests that myoglobin may be a mediator of the progression of metabolic syndrome induced DKD,^24^ but the pathophysiological mechanism of the plasma myoglobin in DKD has not yet been elucidated. Several studies have reported that myoglobin is involved in the pathogenesis of diabetic macrovascular disease.^12^, ^25^ Based the existed evidence, we speculated that myoglobin may be related to DKD. To explore the correlation, we tested two different groups of people with T2DM (DKD and non‐DKD). We then performed case‐control matching analyses and quantile‐stratified cohort studies to determine the association of myoglobin with kidney injury and whether myoglobin helps predict the development of DKD.

## 2 MATERIALS AND METHODS

### 2.1 Study population

The study complied with the Declaration of Helsinki and all subjects gave written informed consent.

The study was approved by the ethics committee of Shanghai Fifth People's Hospital.

**FIGURE 1:**
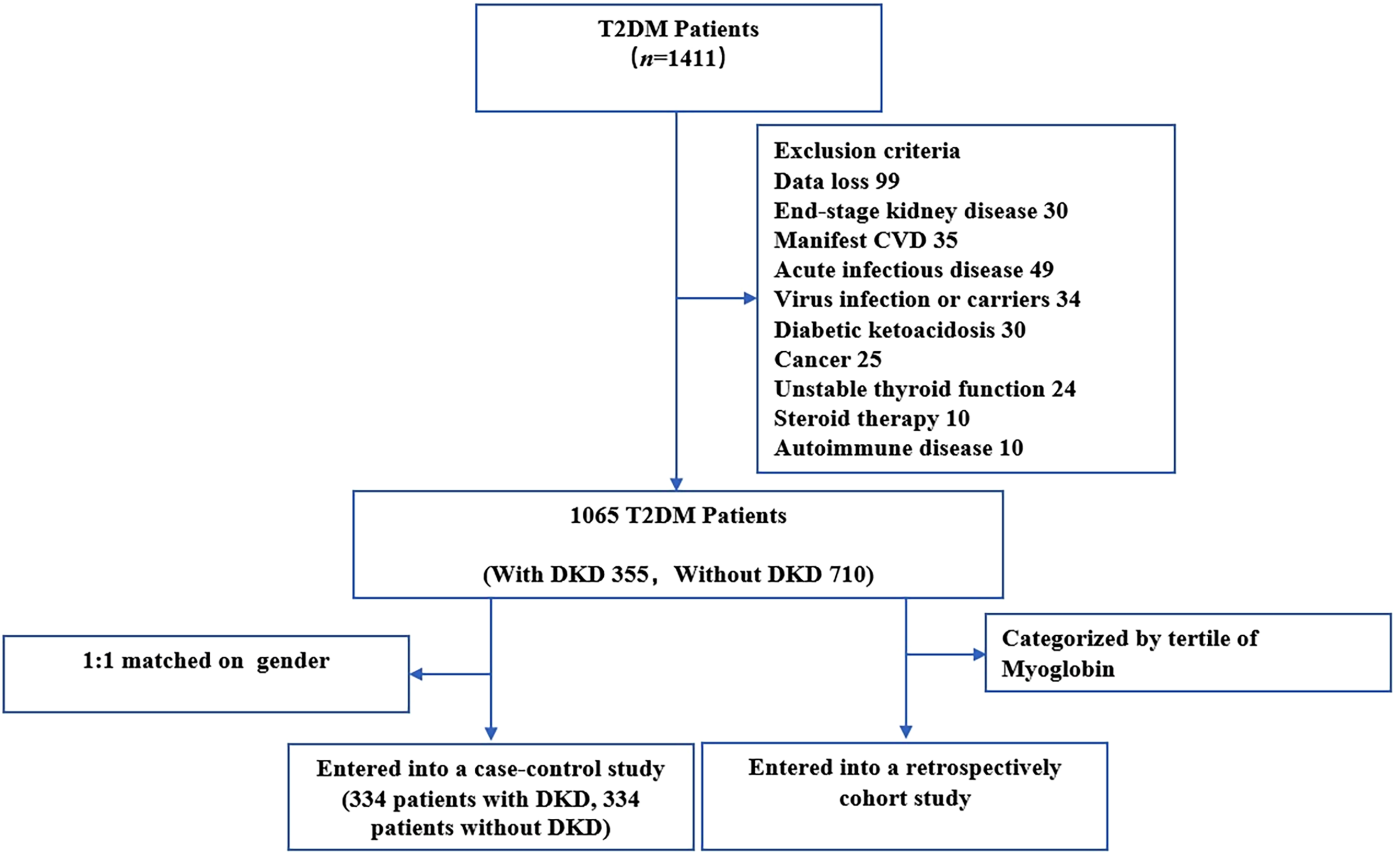
Flow chart of the study. CVD, cardiovascular disease; DKD, diabetic kidney disease; T2DM, type 2 diabetes mellitus.

We strictly followed the DKD diagnostic criteria of the Kidney Disease Outcomes Quality Initiative (KDOQI) clinical practice guideline and excluded other causes of chronic kidney disease during the inclusion process. Using the American Diabetes Association criteria,^26^ 1411 patients with T2DM were recruited in the department of endocrinology of Shanghai Fifth People's Hospital from January 2019 to December 2021. Patients were excluded if they had any of the following: end‐stage kidney disease (defined as receiving dialysis, renal transplantation, or estimated glomerular filtration rate [eGFR] <15 mL/min/1.73 m^2^), manifest cardiovascular disease, acute infectious disease, history of virus infection or carrier status, diabetic ketoacidosis, any kind of cancer, unstable thyroid function, established autoimmune disease, prescribed steroid therapy, or lipid‐lowering agents. The procedure for the retrospective analysis is described in Figure 1. Finally, 355 patients with DKD and 710 patients without DKD were analyzed. DKD was defined according to the diagnostic criteria of the KDOQI clinical practice guideline as the presence of macroalbuminuria, or microalbuminuria in the presence of diabetic retinopathy.^27^ Macroalbuminuria was defined as an albumin to creatinine ratio (ACR) >300 mg/g and microalbuminuria as ACR of 30–300 mg/g in two of three urine samples.

### 2.2 Data collection and laboratory assessments

Patient age and medical history, duration of diabetes, hypertension, body mass index, systolic blood pressure (SBP), and diastolic blood pressure (DBP) were recorded.

After a 12‐h overnight fast, blood was taken for measurement of hemoglobin A1c (HbA1c, Variant II, Bio‐Rad, USA), blood cell count (XN9000, Sysmex, Japan), C‐reactive protein (CRP, XN9000, Sysmex, Japan), biochemical parameters (Cobas 8000, Roche, Switzerland), and myoglobin (Vitros 5600, Johnson, USA). eGFR was calculated using the modification of diet in renal disease equation developed for the Chinese population: eGFR (mL/min/1.73 m^2^) = 186 × (Crea×0.011)^−1.154^ × (age)^−0.203^ × (0.742 if female/1 if male) × 1.233, where creatinine (Crea) is in μmol/L and 1.233 is the adjusting coefficient for Chinese patients.^28^

### 2.3 Statistical analysis

A case–control matching analysis was performed to avoid potential bias due to uneven distribution of covariates between individuals with or without DKD. To further explore the association of biochemical parameters, a cohort study was established. Patients were divided into three groups by tertile of concentration of biochemical parameters that showed a significant difference. Pearson correlation analysis and simple linear regression analysis were used to evaluate the association of biochemical parameters that showed significant changes. To determine the risk factors for development of DKD, binary logistic regression analysis (backward conditional) was used in the matched case–control analysis and the cohort study. Continuous association of plasma myoglobin with DKD incidence was determined by spline regression analysis. Data with a normal distribution are expressed as mean ±SD and were analyzed by Student *t* test or analysis of variance test. Nonnormally distributed variables are expressed as median and interquartile range and were analyzed by nonparametric tests (Mann–Whitney or Kruskal–Wallis test). Categorical variables are presented as frequencies and proportions and were analyzed by *χ* ^2^ test. Statistical descriptions for logistic regression analysis are presented as regression coefficient (SE) and odds ratio (95% confidence interval). All data were analyzed using SPSS 24.0 software (IBM, Armonk, NY) and R software (version R 4.0.1). A two‐tailed *p* value <.05 was considered statistically significant.

## 3 RESULTS

### 3.1 Comparison of clinical characteristics and laboratory parameters in patients with and without DKD in all subjects and in matched case–control study

Demographics and clinical data of all subjects are shown in Table 1. There were significant differences in clinical characteristics of patients with and without DKD including gender (male/female) (355 [84:271] vs 710 [460:250], *p* < .001). Those with DKD tended to be older and have longer disease duration, higher SBP, and lower DBP. Significant differences were also observed in laboratory parameters: including alanine aminotransferase (ALT) that was lower while urea nitrogen (UN), uric acid (UA), and Crea were higher in those with DKD. eGFR was lower and ACR, triglyceride (TG), potassium, and neutrophil count (NEU) were significantly higher and lymphocyte count (LYM) was lower in DKD. NEU to LYM ratio (NLR), CRP, and creatine kinase (CK) were also higher in DKD. Notably, patients with DKD had a significantly higher level of myoglobin than those without (33.4 ± 16.8 vs 55.2 ± 29.6 mg/mL, *p* < .001) (Table 1).

**TABLE 1:** Demographics of the study population.

Variables	All subjects			Matched case–control study		
	Non‐DKD	DKD	*p*	Non‐DKD	DKD	*p*
*n* (male/female)	710 (460: 250)	355 (84: 271)	**<.001**	334 (84: 250)	334 (84: 250)	1.000
Age (years)	59 ± 11	67 ± 8	**<.001**	60 ± 10	67 ± 7	**<.001**
Duration (years)	8.7 ± 7.2	13.4 ± 8.1	**<.001**	9.6 ± 7.3	13.7 ± 8.1	**<.001**
BMI (kg/m^2^)	24.9 ± 3.7	25.4 ± 4.1	.151	24.9 ± 4.1	25.4 ± 4.0	.141
SBP (mm Hg)	130 ± 16	136 ± 20	**<0.001**	131 ± 17	136 ± 18	**.009**
DBP (mm Hg)	80 ± 11	78 ± 12	**.038**	79 ± 11	78 ± 12	.534
HbA1c (%)	9.1 ± 2.3	8.8 ± 2.0	.059	8.8 ± 2.2	8.8 ± 2.0	989
FPG (mmol/L)	7.94 ± 2.81	7.84 ± 3.17	.608	7.80 ± 2.80	7.89 ± 3.17	.709
ALT (U/L)	19.0 (13.0–29.0))	16.0 (11.0–22.0)	**<.001**	19.0 (14.0–29.0)	15.5 (11.0–22.8)	**<.001**
UN (mmol/L)	5.10 ± 1.44	7.03 ± 2.60	**<.001**	4.86 ± 1.31	7.09 ± 2.64	**<.001**
UA (μmol/L)	297 ± 86	350 ± 104	**<.001**	282 ± 85	352 ± 106	**<.001**
Crea (μmol/L)	56 ± 16	92 ± 37	**<.001**	55 ± 14	94 ± 38	**<.001**
eGFR (mL/min/1.73 m^2^)	93.7 ± 24.1	46.9 ± 11.1	**<.001**	82.1 ± 17.5	46.5 ± 11.3	**<.001**
ACR (mg/g)	7.0 (4.0–12.6)	33.0 (6.0–317.0)	**<.001**	7.0 (4.0–12.3)	33.5 (7.0–377.0)	**<.001**
TC (mmol/L)	4.26 ± 1.05	4.36 ± 1.28	.191	4.3 ± 1.1	4.4 ± 1.3	.915
TG (mmol/L)	1.49 (1.07–2.28)	1.82 (1.16–2.44)	**.001**	1.56 (1.10–2.40)	1.79 (1.13–2.44)	**.007**
HDL‐C (mmol/L)	1.08 ± 0.33	1.09 ± 0.32	.541	1.17 ± 0.35	1.09 ± 0.32	**.003**
Potassium (mmol/L)	3.9 ± 0.4	4.1 ± 0.4	**<.001**	3.9 ± 0.3	4.1 ± 0.4	**<.001**
Sodium (mmol/L)	141 ± 2	141 ± 2	.553	141 ± 2	141 ± 2	.733
Magnesium (mmol/L)	0.82 ± 0.07	0.82 ± 0.08	.181	0.81 ± 0.07	0.82 ± 0.09	**.047**
Calcium (mmol/L)	2.26 ± 0.10	2.27 ± 0.14	.065	2.26 ± 0.10	2.27 ± 0.14	.311
Phosphate (mmol/L)	1.16 ± 0.18	1.15 ± 0.17	.282	1.19 ± 0.18	1.14 ± 0.17	.311
NEU (×10^9^/L)	3.60 ± 1.29	3.94 ± 1.40	**<.001**	3.48 ± 1.15	3.94 ± 1.37	**<.001**
LYM (×10^9^/L)	1.84 ± 0.64	1.69 ± 0.68	**.001**	1.80 ± 0.65	1.67 ± 0.67	**<.001**
NLR	2.2 ± 1.2	2.6 ± 1.4	**<.001**	2.2 ± 1.1	2.7 ± 1.4	**<.001**
CRP (mg/L)	2.0 (1.0–4.0)	3.0 (1.0–8.0)	**.001**	2.0 (1.0–3.6)	3.0 (1.0–8.0)	**.002**
CK (U/L)	69 (52–97)	83 (50–112)	**.007**	64 (46–86)	84 (52–113)	**<.001**
Myoglobin (mg/mL)	33.4 ± 16.8	55.2 ± 29.6	**<.001**	31.2 ± 16.6	56.6 ± 29.9	**<.001**

*Note*: Data of normal distribution are expressed as means ± SDs and analyzed by student *t* test. Nonnormally distributed variables are expressed as median and interquartile range, and analyzed by nonparametric test (Mann–Whitney test). Categorical variables are expressed as frequencies and proportions, and analyzed by χ^2^ test. Bold indicates statistical significance (*p* < .05).

Abbreviations: ACR, albumin to creatinine ratio; ALT, alanine aminotransferase; BMI, body mass index; CK, creatine kinase; Crea, creatinine; CRP, C‐reactive protein; DBP, diastolic blood pressure; DKD, diabetic kidney disease; eGFR, estimated glomerular filtration rate; FPG, fasting plasma glucose; HbA1c, hemoglobin A1c; HDL‐C, high‐density lipoprotein cholesterol; LYM, lymphocyte; NEU, neutrophil; NLR, neutrophil to lymphocyte ratio; SBP, systolic blood pressure; TC, total cholesterol; TG, triglyceride; UA, uric acid; UN, urea nitrogen.

To avoid statistical bias that could cause uneven distribution of covariates between individuals with and without DKD, a 1:1 case–control matching analysis was performed. After covariate matching, similar significant differences remained for age, disease duration, SBP, ALT, UN, UA, Crea, eGFR, ACR, TG, potassium, NEU count, LYM count, NLR, CRP, CK, and myoglobin (Table 1). In addition, significant differences emerged in high‐density lipoprotein cholesterol (HDL‐C) and magnesium, respectively lower and higher in those with DKD (Table 1).

### 3.2 Elevated plasma myoglobin as an independent risk factor for DKD in the matched casecontrol study

To further determine whether myoglobin could be considered a risk factor for DKD, binary logistic regression analysis with backward conditional selection was performed in the matched case–control study. Included patients were divided into different groups by age, disease duration, SBP, ALT, TG, HDL‐C, potassium, magnesium, tertile of NLR (lowest group <1.8, middle group [1.8–2.5], and highest group >2.5), tertile of CRP (lowest group <1.3 mg/L, middle group [1.3–4.0 mg/L], and highest group >4.0 mg/L), tertile of CK (lowest group <61 U/L, middle group [61–91 U/L], and highest group >92 U/L), and tertile of myoglobin (lowest group <28.1 mg/mL, middle group 28.1–45.6 mg/mL, and highest group >45.6 mg/mL). Myoglobin showed a significantly higher odds ratio (OR) value in the middle tertile (OR: 7.414; 95% confidence interval [CI]: 2.301–23.888; vs the lowest tertile, *p* = .001) and the highest tertile (OR: 13.500; 95% CI: 3.788–48.113; vs the lowest tertile, *p* < .001).

Significant differences were also observed for age (OR: 1.121; 95% CI: 1.040–1.208, *p* = .003), HDL‐C (OR: 6.678; 95% CI: 1.833–24.323; vs Reference, *p* = .004) and magnesium (OR: 6.772; 95% CI: 1.279–35.859; vs Reference, *p* = .024) (Table 2).

**TABLE 2:** Binary logistic regression analysis (backward conditional) to determine the risk factors for development DKD in the matched case–control study.

Variables	*β* (SE)	OR (95% CI)	*p*value
Age (years)	0.114 (0.038)	1.121 (1.040–1.208)	**.003**
Duration (years)	0.061 (0.034)	1.062 (0.994–1.135)	.074
HDL‐C (mmol/L)			
≥1.0 (M) or ≥1.3 (F)	Reference	Reference	
<1.0 (M) or <1.3 (F)	1.899 (0.660)	6.678 (1.833–24.323)	**.004**
Potassium (mmol/L)			
<4.2	Reference	Reference	
≥4.2	1.065 (0.549)	2.900 (0.989–8.500)	.052
Magnesium (mmol/L)			
<0.90	Reference	Reference	
≥0.90	1.913 (0.850)	6.772 (1.279–35.859)	**.024**
Tertile of myoglobin (mg/mL)			
Lowest	Reference	Reference	
Middle	2.003 (0.597)	7.414 (2.301–23.888)	**.001**
Highest	2.603 (0.648)	13.500 (3.788–48.113)	**<.001**

*Note*: Data are presented as regression coefficient (SE), odds ratio (95% confidence interval), and *p* value. Binary logistic regression analysis (backward conditional) was used determine the risk factors for DKD in the matched case–control study. Bold indicates statistical significance (*p* < .05).

Abbreviations: CI, confidence interval; DKD, diabetic kidney disease; HDL‐C, high‐density lipoprotein cholesterol; OR odds ratio.

### 3.3 Comparison of clinical parameters among three groups categorized by tertile of plasma myoglobin in the cohort study

Subjects were divided into three groups according to tertile of myoglobin, lowest group (below 28.1 mg/mL), middle group (28.1–45.6 mg/mL), and highest group (above 45.6 mg/mL) (Table 3). Significant differences were observed for gender (male/female) (222 [21:201] vs 223 [48:175] vs 223 [99:124], *p* < .001) and for age, disease duration, SBP, UN, UA, and Crea, all of which increased from the lowest to middle and highest tertile whereas eGFR decreased. ACR, potassium, and magnesium likewise significantly increased across the three tertiles whereas HDL‐C increased across the lowest to middle tertile. Phosphate decreased and NEU increased, LYM decreased and corresponding NLR increased as did high‐sensitivity CRP and CK across the lowest, middle, and highest groups (Table 3).

**TABLE 3:** Comparison of clinical parameters among three groups categorized by tertile of myoglobin in the cohort study.

Variables	Lowest group	Middle group	Highest group	*p* value
Myoglobin range (mg/mL)	below 28.1	28.1–45.6	above 45.6	
*n* (male/female)	222 (21: 201)	223 (48: 175)	223 (99: 124)	**<.001**
Age (years)	58 ± 10	66 ± 8	67 ± 7	**<.001**
Duration (years)	9.6 ± 7.1	12.2 ± 8.2	13.4 ± 8.2	**.001**
BMI (kg/m^2^)	24.9 ± 4.1	25.2 ± 4.0	25.4 ± 4.0	.825
SBP (mmHg)	130 ± 17	133 ± 17	137 ± 21	**.010**
DBP (mmHg)	79 ± 11	78 ± 11	79 ± 11	1.000
HbA1c (%)	8.8 ± 2.1	8.9 ± 2.1	8.8 ± 2.1	1.000
FPG (mmol/L)	7.78 ± 2.76	8.24 ± 3.09	7.51 ± 3.07	1.000
ALT (U/L)	16.0 (12.0–29.0)	18.0 (14.0–28.0)	15.0 (11.0–21.5)	1.000
UN (mmol/L)	4.81 ± 1.36	5.50 ± 1.50	7.60 ± 2.90	**<.001**
UA (μmol/L)	282 ± 82	295 ± 96	372 ± 105	**<.001**
Crea (μmol/L)	54 ± 12	67 ± 19	104 ± 41	**<.001**
eGFR (mL/min/1.73 m^2^)	77.4 ± 18.9	65.6 ± 22.0	49.9 ± 19.6	**<.001**
ACR (mg/g)	6.0 (4.0–14.0)	10.0 (5.0–24.0)	62.8 (9.5–810.7)	**<.001**
TC (mmol/L)	4.44 ± 1.10	4.28 ± 1.10	4.33 ± 1.38	1.000
TG (mmol/L)	2.02 (1.29–2.69)	1.49 (1.03–2.42)	1.52 (1.14–2.21)	1.000
HDL‐C (mmol/L)	1.15 ± 0.36	1.18 ± 0.33	1.05 ± 0.31	**.007**
Potassium (mmol/L)	3.9 ± 0.3	4.0 ± 0.4	4.1 ± 0.5	**.001**
Sodium (mmol/L)	141 ± 2	141 ± 2	141 ± 2	1.000
Magnesium (mmol/L)	0.81 ± 0.07	0.81 ± 0.07	0.83 ± 0.09	**.004**
Calcium (mmol/L)	2.26 ± 0.10	2.27 ± 0.10	2.27 ± 0.15	1.000
Phosphate (mmol/L)	1.22 ± 0.17	1.15 ± 0.18	1.13 ± 0.18	**<.001**
NEU (×10^9^/L)	3.44 ± 1.20	3.67 ± 1.14	4.00 ± 1.43	**<.001**
LYM (×10^9^/L)	1.86 ± 0.69	1.75 ± 0.61	1.59 ± 0.67	**<.001**
NLR	2.1 ± 1.1	2.3 ± 1.1	2.9 ± 1.5	**<.001**
CRP (mg/L)	2.0 (1.0–3.8)	2.0 (1.0–5.0)	2.5 (1.0–8.0)	**.047**
CK (U/L)	50 (10–69)	74 (53–92)	102 (81–130)	**<.001**

*Note*: Data with normal distribution are expressed as mean ± SD and analyzed by analysis of variance test. Nonnormally distributed variables are expressed as median and interquartile range and analyzed by nonparametric test (Kruskal–Wallis). Categorical variables are expressed as frequencies and proportions, and analyzed by *χ* ^2^ test. Bold indicates statistical significance (*p* < .05).

Abbreviations: ACR, albumin to creatinine ratio; ALT, alanine aminotransferase; BMI, body mass index; CK, creatine kinase; Crea, creatinine; CRP, C‐reactive protein; DBP, diastolic blood pressure; eGFR, estimated glomerular filtration rate; FPG, fasting plasma glucose; HbA1c, hemoglobin A1c; HDL‐C, high‐density Lipoprotein cholesterol; LYM, lymphocyte; NEU, neutrophil; NLR, neutrophil to lymphocyte ratio; SBP, systolic blood pressure; TC, total cholesterol; TG, triglyceride; UN, urea nitrogen; UA, uric acid.

### 3.4 Plasma myoglobin was closely associated with renal function and inflammatory parameters

**FIGURE 2:**
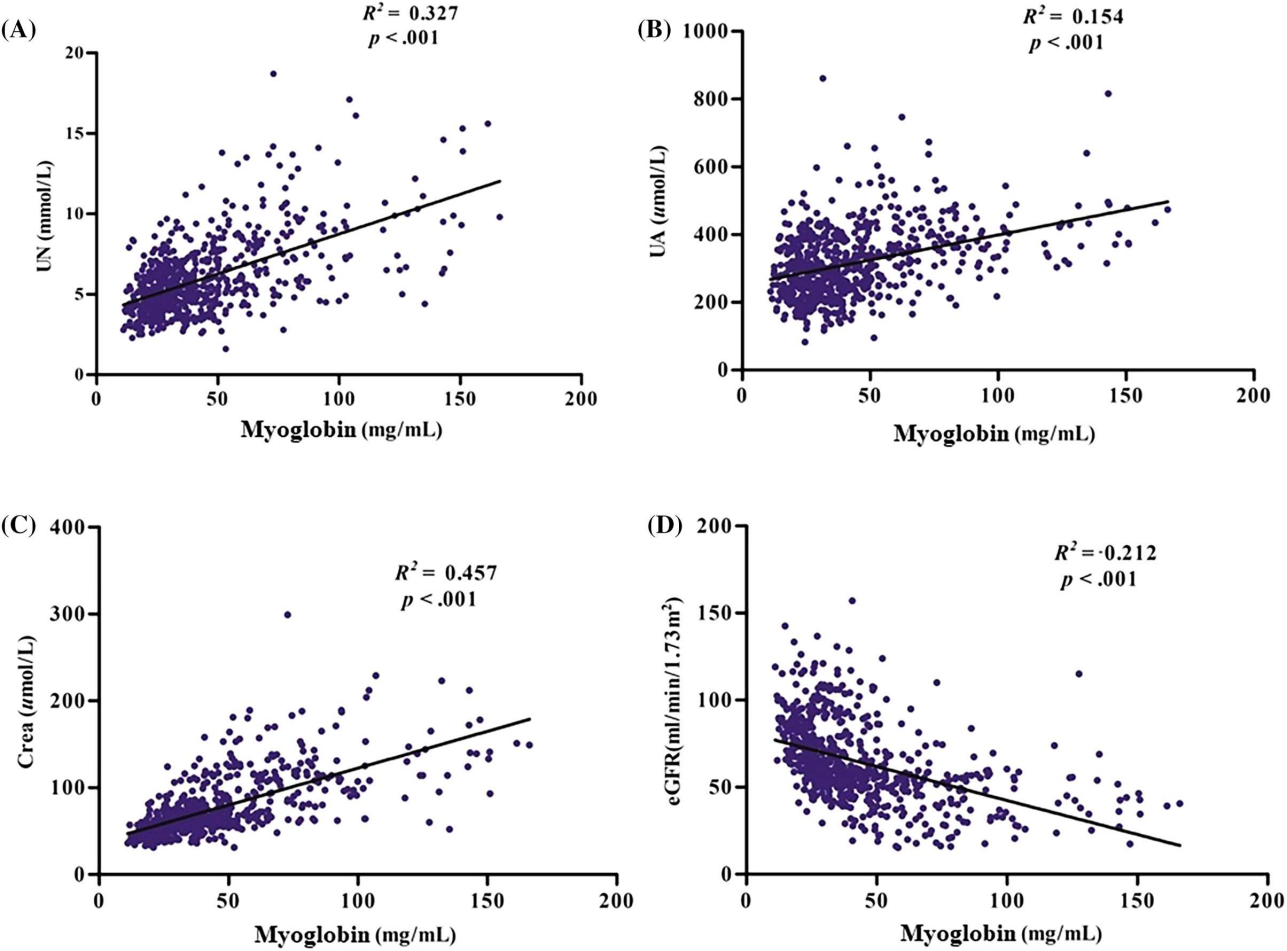
Simple linear regression analysis between plasma myoglobin and renal function. Plasma myoglobin was positively associated with UN (A), UA (B), and Crea (C). Meanwhile, plasma myoglobin was negatively associated with eGFR. Crea, creatinine; eGFR, estimated glomerular filtration rate; UA, uric acid; UN, urea nitrogen.

**FIGURE 3:**
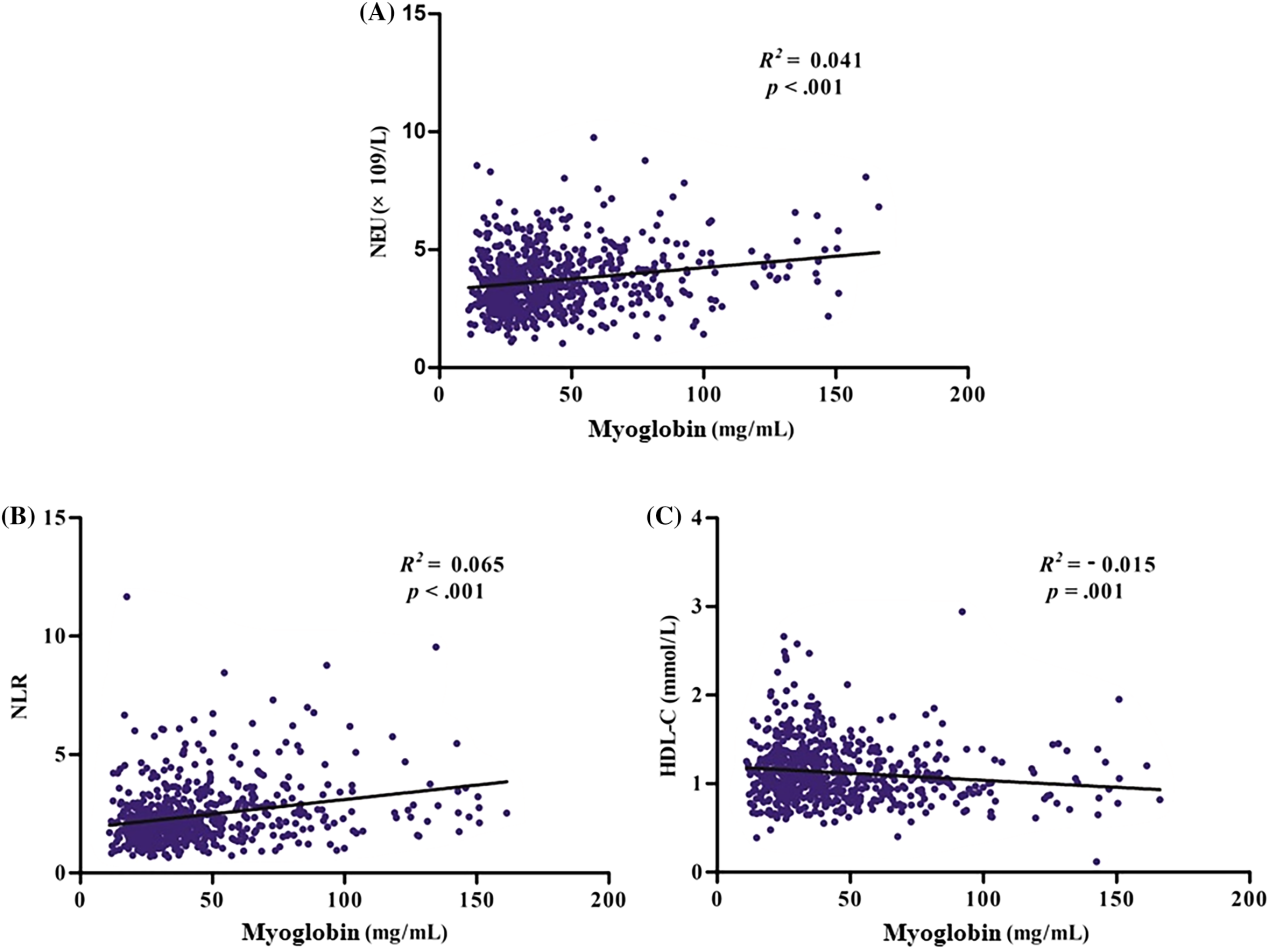
Simple linear regression analysis between plasma myoglobin and inflammatory markers. Myoglobin was positively associated with NEU count (A) and NLR (B), and negatively associated with HDL‐C (C). NEU, neutrophil; NLR, neutrophil‐to‐lymphocyte ratio; HDL‐C, high‐density lipoprotein cholesterol.

To further investigate the correlation between these significant differences and progression of DKD, correlation analysis was performed. Pearson correlation analysis revealed that myoglobin was positively and significantly correlated with UN (Figure S1A), UA (Figure S1B), Crea (Figure S1C), NEU count (Figure S2A), and NLR (Figure S2B) and negatively associated with eGFR (Figure S1D) and HDL‐C (Figure S2C), all at *p* < .001. Simple linear regression analysis revealed that myoglobin was likewise positively and significantly associated with UN (Figure 2A), UA (Figure 2B), Crea (Figure 2C), NEU count (Figure 3A), and NLR (Figure 3B) and negatively associated with eGFR (Figure 2D) and HDL‐C (Figure 3C).

### 3.5 Elevated plasma myoglobin was an independent risk factor for the development of DKD in the cohort study

In the cohort study, to determine independent risk factors for the development of DKD, tertile of myoglobin, age, HDL‐C, potassium, magnesium, and gender were entered into binary logistic regression analysis with backward conditional selection. Plasma myoglobin showed a significantly higher OR value in the middle tertile (OR: 7.060; 95% CI: 2.244–22.211; vs the lowest tertile, *p* = .001) and the highest tertile (OR: 21.965; 95% CI: 5.269–91.578; vs the lowest tertile, *p* < .001). Age (OR: 1.123; 95% CI: 1.045–1.208, *p* = .002), HDL‐C (OR: 7.434; 95% CI: 2.106–26.244; vs Reference, *p* = .002), potassium (OR: 3.083; 95% CI: 1.084–8.768 vs Reference, *p* = .035), and magnesium (OR: 5.351; 95% CI: 1.194–23.972; vs Reference, *p* = .028) also showed significant differences (Table 4).

**TABLE 4:** Binary logistic regression analysis (backward conditional) to determine the risk factors for development of DKD in the cohort study.

Variables	*β* (SE)	OR (95% CI)	*p* value
Tertile of myoglobin (mg/mL)			
Lowest	Reference	Reference	
Middle	1.954 (0.585)	7.060 (2.244–22.211)	**.001**
Highest	3.089 (0.728)	21.965 (5.269–91.578)	**<.001**
Age (years)	0.116 (0.037)	1.123 (1.045–1.208)	**.002**
HDL‐C (mmol/L)			
≥1.0 (M) or ≥1.3 (F)	Reference	Reference	
<1.0 (M) or <1.3 (F)	2.006 (0.644)	7.434 (2.106–26.244)	**.002**
Potassium (mmol/L)			
<4.2	Reference	Reference	
≥4.2	1.126 (0.533)	3.083 (1.084–8.768)	**.035**
Magnesium (mmol/L)			
<0.90	Reference	Reference	
≥0.90	1.677 (0.765)	5.351 (1.194–23.972)	**.028**
Gender			
Male	Reference	Reference	
Female	1.144 (0.617)	3.140 (0.937–10.517)	.064

*Note*: Data are presented as regression coefficient (SE), odds ratio (95% confidence interval), and *p* value. Logistic regression analysis (backward conditional) was used determine the risk factors for development of DKD in the cohort study. Bold indicates statistical significance (*p* < .05).

Abbreviations: CI, confidence interval; DKD, diabetic kidney disease; HDL‐C, high‐density lipoprotein cholesterol; OR odds ratio.

### 3.6 The accuracy of plasma myoglobin for the diagnosis of DKD

**FIGURE 4:**
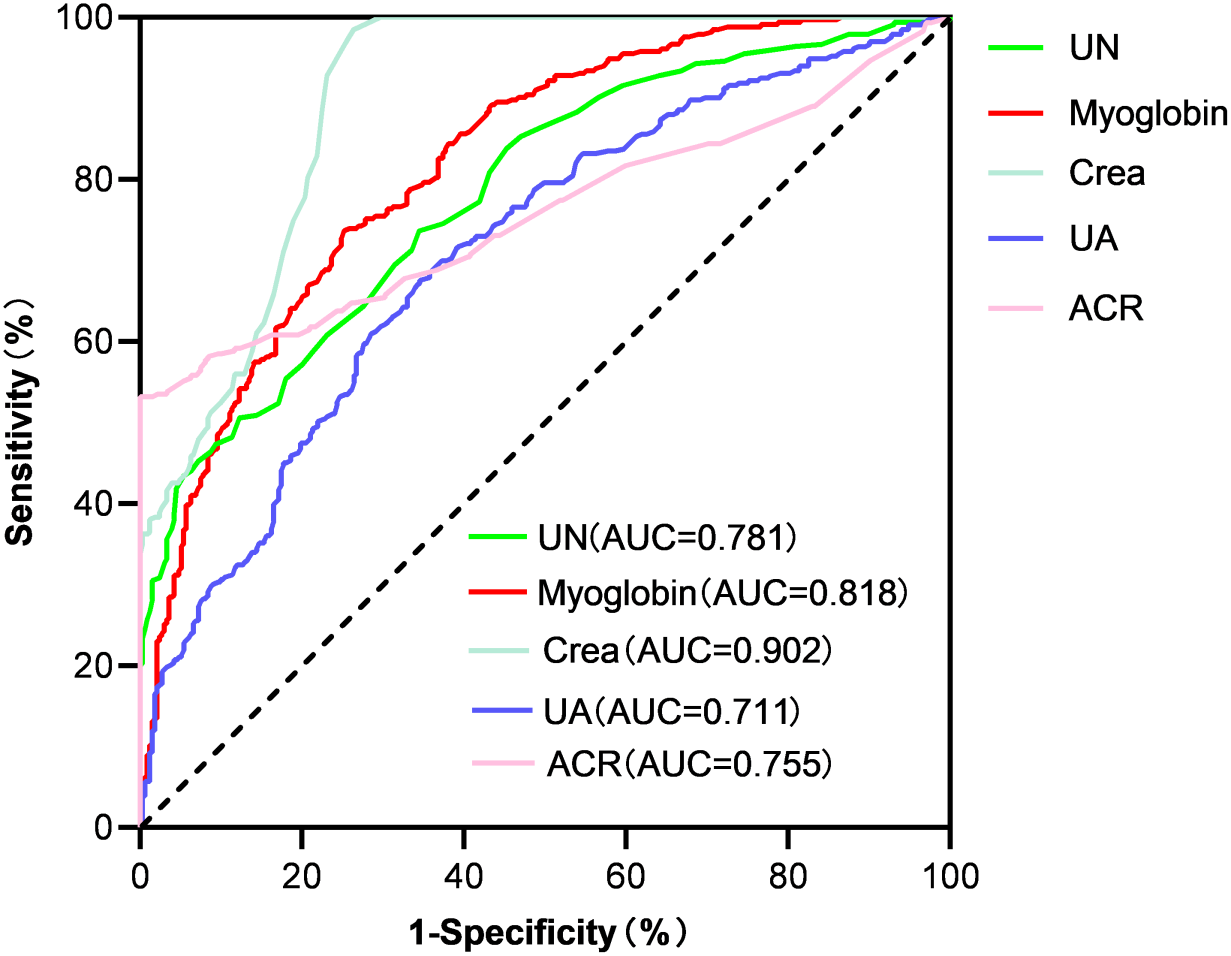
ROC curve of plasma myoglobin and related indicators in the diagnosis of DKD. ACR, albumin creatinine ratio; AUC, area under ROC curve; Crea, creatinine; DKD, diabetic kidney disease; ROC, receiver operating characteristics; UA, uric acid; UN, urea nitrogen.

The area under the receiver operating characteristics curves of myoglobin, blood urea nitrogen, Crea, UA, and ACR were 0.818 (95% CI: 0.787–0.860, *p* < .001), 0.781 (95% CI: 0.745–0.816, *p* < .001), 0.902 (95% CI: 0.879–0.925, *p* < .001), 0.711 (95% CI: 0.671–0.751, *p* < .001), and 0.755 (95% CI: 0.715–0.795, *p* < .001), respectively. The sensitivity, specificity, and cutoff values of myoglobin were evaluated. Plasma myoglobin has important diagnostic value in the diagnosis of DKD. The cutoff value with the highest Youden index (0.496) was defined as the optimum. The optimal value of plasma myoglobin as an indicator for monitoring the development of DKD was 36.1 mg/mL, yielding a sensitivity of 73.7% and specificity of 74.9% (Figure 4).

### 3.7 Continuous plasma myoglobin was closely associated with the incidence of DKD

**FIGURE 5:**
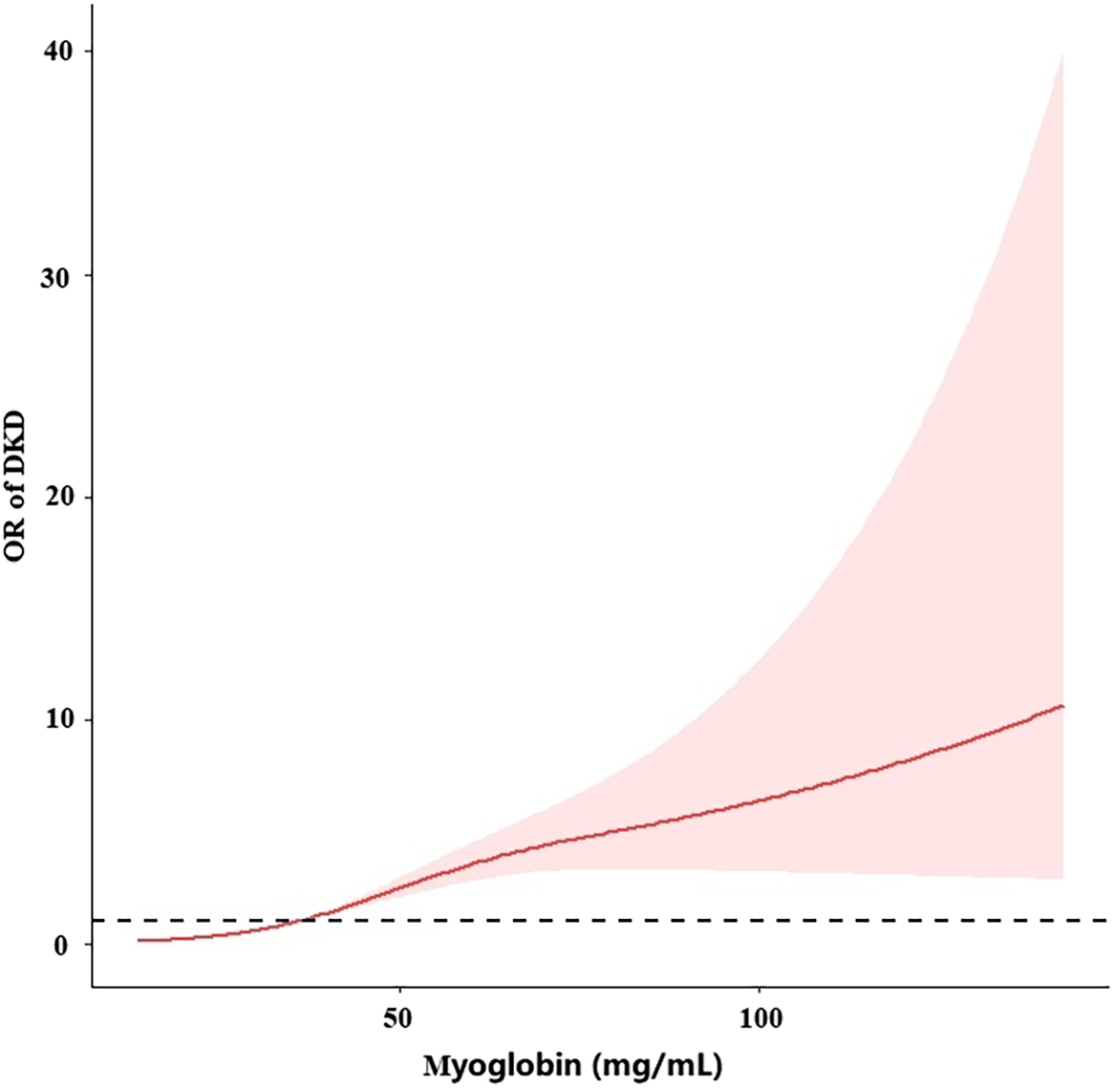
Continuous association of plasma myoglobin with the incidence of DKD. Adjusted for age, HDL‐C, and magnesium. DKD, diabetic kidney disease; HDL‐C, high‐density lipoprotein cholesterol; OR, odds ratio.

After adjusting for age, HDL‐C, and magnesium, a spline model showed a significant relationship between continuous myoglobin and DKD incidence. The risk of developing DKD increased rapidly when myoglobin exceeded 36.4 mg/mL (Figure 5).

## 4 DISCUSSION

To date, a large number of studies have been devoted to screening the biomarkers associated with kidney injury in diabetic patients. In this study, we found that a significant high level of plasma myoglobin was observed in DKD. Plasma myoglobin had a strong linear correlation with renal function and inflammatory related markers, and the higher myoglobin level was significantly associated with increased risk of renal injury in T2DM patients. Thus, further sensitivity analysis of the myoglobin data showed that plasma myoglobin could be used to monitor the development of DKD. Myoglobin is an iron‐containing protein, synthesized in cardiomyocytes and skeletal muscle cells, and plays an important role in the storage and transport of molecular oxygen for cellular respiration.^6^, ^7^, ^8^, ^9^, ^10^, ^11^ Myoglobin has a higher oxygen binding capability than hemoglobin, enabling it to store oxygen and release it during periods of deprivation in muscle tissue. The physiological processes in which myoglobin participates in oxygen deposition and diffusion may involve mitochondrial metabolism and extend physiological process.^29^ Myoglobin is also an antioxidant agent that protects cells by ROS elimination.^15^, ^16^ Myoglobin may play a role in fatty acid metabolism, based on strong evidence that various myoglobin ligand forms can bind fatty acids.^30^, ^31^

As an important risk factor for DKD, the pathogenesis of myoglobin in DKD remains unclear. The pathophysiology of myoglobin has been widely studied in rhabdomyolysis, a condition consequent to muscle injury. It is usually associated with trauma but can also develop in hyperthermia, muscle ischemia, or during seizures. As a consequence of muscle breakdown, myoglobin is released into the circulation and deposited in kidney tissue with consequent acute kidney injury (AKI).^32^ In recent years, Ruoru Wu et al have found that serum myoglobin as a mediator of metabolic syndrome induced renal impairment, consistent with low HDL‐C as a risk factor for DKD in our study.^24^ A study on diabetic nephropathy showed a strong correlation between serum myoglobin and DKD.^33^ In another study, elevated serum myoglobin level was associated with advanced chronic kidney disease.^34^ We speculate that myoglobin may be closely related to DKD. Our study confirms our hypothesis that myoglobin is associated with renal function. Myoglobin is an independent risk factor for DKD. Moreover, fully adjusted spline regression shows a significant correlation of continuous myoglobin with DKD incidence with an abrupt increase in risk when myoglobin exceeded 36.4 mg/mL.

In addition, myoglobin could promote oxidation of ferrous (Fe^2+^) to ferric iron (Fe^3+^) form in tubular cells, inducing lipid peroxidation and malondialdehyde synthesis.^35^, ^36^, ^37^ This increased accumulation of ROS derived from iron metabolism can induce oxidative damage to cell membranes and threaten cell integrity and survival. This form of regulated nonapoptotic cell death is termed ferroptosis.^38^ Ferroptosis has been implicated in multiple pathological conditions including acute renal failure and ischemia/reperfusion injury.^39^, ^40^ Meanwhile, amplification of ROS can also activate caspases‐1 and caspases‐3 to induce tubular cell apoptosis by mitogen‐activated protein kinase (MAPK) signaling.^41^, ^42^, ^43^ On the other hand, proinflammatory mediators, including chemokines, cytokines, and inflammasomes released by endothelial and tubular cells, have been reported to contribute to kidney inflammation and injury during the early phase of rhabdomyolysis‐induced AKI.^44^, ^45^, ^46^ Our study confirms that myoglobin is positively correlated with NEU and NLR and negatively correlated with HDL‐C.

In light of these findings, we propose that myoglobin can help predict kidney injury in patients with T2DM and serve as a supplementary biomarker to evaluate the development of established DKD. Myoglobin may also be a biomarker to reflect the level of ferroptosis in the kidney that may be indirectly correlated with the loss of the nephrons in DKD. Thus, myoglobin offers a more accurate means by which to monitor kidney injury than eGFR loss and albuminuria, particularly when eGFR does not reach the threshold of kidney injury during early stage DKD.

Some limitations could not be avoided. The cross‐sectional method prevented exploration of a causal relationship between myoglobin and DKD. Future longitudinal studies may provide clarification. In conclusion, this study identifies novel evidence that the level of plasma myoglobin is significantly higher in patients with DKD and shows a significant linear association with renal function. As an independent risk factor, the evaluation of myoglobin may be used in daily clinical practice to predict the development of DKD. Plasma myoglobin is an independent risk factor for DKD development when it exceeds 36.4 mg/mL.

## FUNDING INFORMATION

This work was partially supported by the Science Foundation of Shanghai Fifth People's Hospital (No. 2018WYZT03), Fudan University‐Minhang Health Consortium Cooperation Fund Project (No. 2022FM01), Community Health Research Project of Community Health Research Center of Fudan University (No. 2020SJ06), and Kaihua County Doctoral Workstation Fund of Zhejiang Province (No. 2023KH02).

## CONFLICT OF INTEREST STATEMENT

The authors declare that there is no conflict of interest that could be perceived as prejudicing the impartiality of the research reported.

## Supporting information


**FIGURE S1.** Pearson correlation analysis between plasma myoglobin and renal function. Crea, creatinine; eGFR, estimated glomerular filtration rate; UA, uric acid; UN, urea nitrogen.

**FIGURE S2.** Pearson correlation analysis between plasma myoglobin and inflammatory markers. HDL‐C, highdensity lipoprotein cholesterol; NEU, neutrophil; NLR, neutrophil‐to‐lymphocyte ratio.
