# Supplementary figures and images for: Elevated plasma myoglobin level is closely associated with type 2 diabetic kidney disease
Source: J Diabetes. 2023 Nov 30;16(3):e13508. doi: 10.1111/1753-0407.13508 (PMC10925879; doi:10.1111/1753-0407.13508)

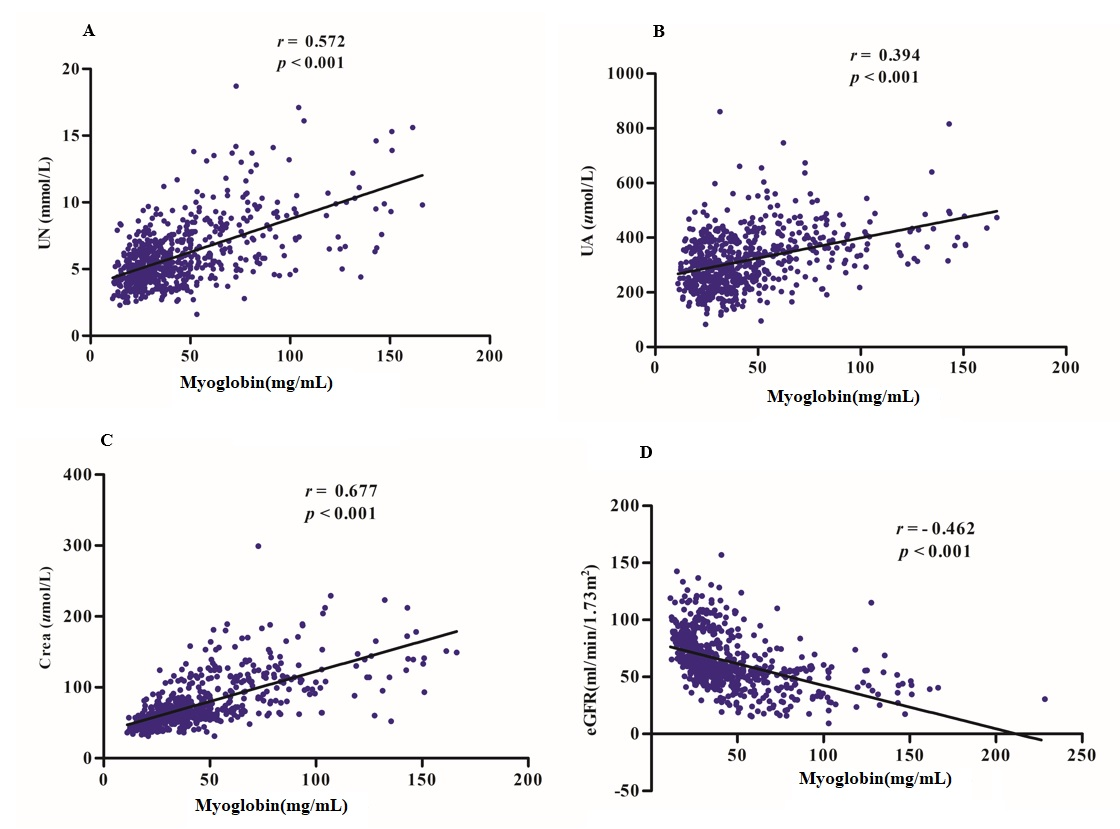

Supplement: Supplementary file 1 — FIGURE S1. Pearson correlation analysis between plasma myoglobin and renal function. Crea, creatinine; eGFR, estimated glomerular filtration rate; UA, uric acid; UN, urea nitrogen. [file JDB-16-e13508-s002.tif]

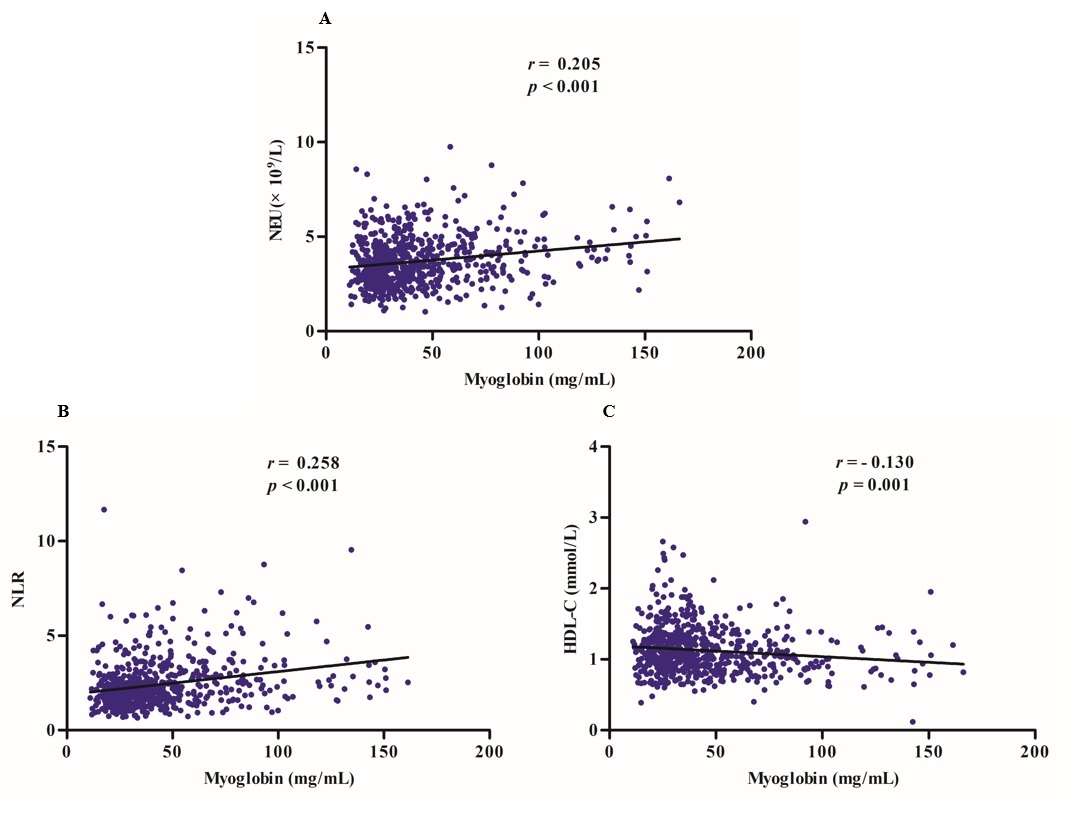

Supplement: Supplementary file 2 — FIGURE S2. Pearson correlation analysis between plasma myoglobin and inflammatory markers. HDL‐C, highdensity lipoprotein cholesterol; NEU, neutrophil; NLR, neutrophil‐to‐lymphocyte ratio. [file JDB-16-e13508-s001.tif]
